# Clinical effects of debulking surgery combined with neoadjuvant chemotherapy in treating ovarian cancer and its influence on tumor markers and immune function

**DOI:** 10.12669/pjms.40.11.9337

**Published:** 2024-12

**Authors:** Huijuan Yan, Weiwei Wang, Shengchao Wang, Chunmei Yan, Li Zhu

**Affiliations:** 1 Huijuan Yan, Department of Gynecology, Yellow River Sanmenxia Hospital, Sanmenxia 472001, Henan, China; 2 Weiwei Wang, Department of Gynecology, Yellow River Sanmenxia Hospital, Sanmenxia 472001, Henan, China; 3 Shengchao Wang Department of Thyroid Surgery, Yellow River Sanmenxia Hospital, Sanmenxia 472001, Henan, China; 4 Chunmei Yan, Department of Hematology and Oncology, The 988th Hospital of the Joint Logistics Support Force of the People’s Liberation Army of China, Zhengzhou 450000, Henan, China; 5 Li Zhu Department of Gynecology, Yellow River Sanmenxia Hospital, Sanmenxia 472001, Henan, China

**Keywords:** Ovarian Cancer, Debulking Surgery, Neoadjuvant Chemotherapy, Tumor Markers, Immune Function, Quality of Life

## Abstract

**Objective:**

Exploring the clinical efficacy of tumor cell reduction combined with neoadjuvant chemotherapy in treatment of ovarian cancer

**Methods:**

Retrospective analysis was conducted of one hundred patients with advanced ovarian cancer at Yellow River Sanmenxia Hospital from January 2017 to December 2022. They were divided into control group and observation group based on treatment methods, with 50 cases in each group. The control group was treated with tumor cell reduction surgery, the observation group treated with neoadjuvant chemotherapy and tumor cell reduction surgery. The surgical related conditions, clinical treatment effects, tumor markers, cellular immune function levels, quality of life, and adverse reactions were compared.

**Results:**

The observation group had shorter surgical and hospitalization times, with less intraoperative blood loss and abdominal fluid accumulation compared to control group (P<0.05). The serum levels in both groups of patients were significantly reduced, and observation group had a higher degree of decrease (P<0.05). The levels of cellular immune function levels in both groups improved significantly, and observation group showed more significant improvement (P<0.05). The total efficacy of observation group patients was 72.00%, higher than control group’s 52.00% (P<0.05). The SS-QOL scores of both groups of patients improved significantly, and the degree of improvement in observation group was significant (P<0.05).

**Conclusion:**

The combination of tumor cell reduction surgery and neoadjuvant chemotherapy for ovarian cancer can significantly shorten surgical and hospitalization time, inhibit tumor marker expression, improve patient immune function and quality of life.

## INTRODUCTION

Ovarian cancer is a common malignant tumor disease in gynecology, mostly occurring in middle-aged and elderly women.^1^ Its early symptoms are often not obvious, most patients are diagnosed in the middle and late stage, and surgery combined with chemotherapy is necessary for these patients.^2^ At present, it is advocated to adopt primary tumor cell depletion surgery (PDS) for the treatment of ovarian cancer.^3^ However, for ovarian cancer patients with large tumor volume and advanced stage, the effect of PDS alone is not ideal. Neoadjuvant chemotherapy has been applied in the treatment of ovarian cancer.^4^ By reducing the volume of the primary tumor lesion through chemotherapy, it can reduce tumor burden and staging, improve patient tolerance, enhance surgical treatment effectiveness, improve patient prognosis and survival time. Based on this, this study explored the clinical efficacy of tumor cell reduction combined with neoadjuvant chemotherapy in the treatment of ovarian cancer and its impact on tumor markers and immune function in patients.

## METHODS

A retrospective analysis was conducted on the clinical data of one hundred patients with advanced ovarian cancer admitted to Yellow River Sanmenxia Hospital from January 2017 to December 2022. The patients were divided into the control group(n=50) and the observation group (n=50) according to treatment methods.

### Ethical Approval:

The study was approved by the Institutional Ethics Committee of Yellow River Sanmenxia Hospital (No.: 2021-0901; date: September 01, 2021), and written informed consent was obtained from all participants.

### Inclusion criteria:

- Patients were diagnosed with ovarian cancer by clinical examination and in clinical stage ≥ III;
- Patients with first-time or initial treatment;
- Patients with an expected survival time≥6 months;
- Patients with complete clinical and follow-up data.

### Exclusion criteria:

- Patients with critical concomitant organ diseases and significant liver and kidney dysfunction;
- Patients with mental disorders or unstable vital signs;
- Patients combined with other malignant tumors;
- Patients with incomplete clinical data;
- Patients with previous history of ovarian or uterine surgery.

### Chemotherapy Methods:

***Observation Group:*** two-three cycles of neoadjuvant chemotherapy were performed preoperatively. PT regimen: Intravenous infusion of 75mg/m^2^ cisplatin + 135mg/m^2^ paclitaxel, with a treatment cycle of 21 days. If the PT regimen is ineffective, the PC regimen was adopted: Intravenous infusion of 75 mg/m^2^ cisplatin + 750 mg/m^2^ cyclophosphamide, with a treatment cycle of 21 days. two-three weeks after the completion of chemotherapy, the PDS was performed. Control group: A conventional chemotherapy regimen was applied, and intraperitoneal cisplatin combined with paclitaxel was administered as routine chemotherapy after PDS. four weeks later, an intravenous infusion of 125-175 mg/m^2^ paclitaxel was given, with a treatment cycle of 21 days. Patients in both groups underwent chemotherapy for six months post-operation.

### Surgical Methods for PDS:

The surgery was performed under general anesthesia. If ascites were present after the incision of the peritoneum, it was submitted for a cytological examination. If no ascites, a physiological saline solution (100-200 mL) was used to flush the grooves on both sides of the colon, followed by a complete abdominal exploration to identify the extent of organ involvement and tumor infiltration. Afterward, the greater omentum, uterus and adnexa, pelvic masses, and metastatic lesions were removed before performing the lymph node dissection. For larger tumors, the uterus and adnexa could be removed first, followed by the greater omentum, and the maximum residual

- Operation time, intraoperative blood loss, volume of abdominal effusion, and length of hospital stay.
- Use enzyme-linked immunosorbent assay to detect serum carbohydrate antigen 125 (CA125), serum carbohydrate antigen 153 (CA153), and human epididymal protein 4 (HE4) in both groups of patients before and 6 months after treatment.
- Fasting venous blood samples were collected from both groups pre-treatment and six months post-treatment. Meanwhile, peripheral blood T lymphocyte subsets.
- The Response Evaluation Criteria in Solid Tumors (RECIST) was utilized to evaluate the clinical efficacy, which was divided into complete response (CR), partial response (PR), stable disease (SD) and prog
- The quality of life of patients was assessed using the Short-Form Quality of Life Questionnaire (SS-QOL) pre-treatment and six months post-treatment.
- ***Adverse reactions:*** The adverse reactions due to treatment were evaluated using the National Cancer Institute Common Toxicity Criteria 3.0 (NCI CTCAE 3.0).

### Statistical Methods:

SPSS 21.0 software was used for statistical analysis. Measurement data were expressed as mean ± standard deviation (*χ̅*±*S*), with the independent sample t-test used for comparison between groups. Meanwhile, count data were expressed as the number of cases and percentage (%), and the chi-square test was used for comparison between groups. P<0.05 was considered statistically significant.

## RESULTS

There was no significant difference in general data between the two groups (P > 0.05). Compared with the control group, the observation group significantly shorter operation time and hospital stay and significantly less intraoperative blood loss and abdominal effusion (P < 0.05). Table-I.

**Table-I:** Comparison of Surgical Indicators between the 2 Groups (*χ̅*±*S*).

Group	Operation time (min)	Intraoperative blood loss (mL)	Abdominal effusion (mL)	Hospital stay (d)
Observation Group	172.30±8.99	644.36±58.23	189.30±1.37	11.36±1.68
Control Group	225.60±7.26	739.46±34.17	376.28±2.31	16.44±2.41
*t*	32.617	9.961	491.497	12.243
*P*	<0.001	<0.001	<0.001	<0.001

The post-treatment levels of serum CA125, CA153, and HE4 in two groups were significantly lower than pre-treatment, with significant decrease seen in the observation group than control group (P < 0.05). The post-treatment levels of CD3+, CD4+, and CD4+/CD8+ in two groups were significantly improved compared with the pre-treatment, with the observation group demonstrating more significant improvement than control group (P < 0.05). Table-II. The OR rate in the observation group was 72.00%, higher than control group (52.00%) (P < 0.05). Table-III.

**Table-II:** Comparison of Pre-and Post-Treatment Levels of Tumor Markers and Immune Function between the 2 Groups (*χ̅*±*S*).

Indicator	Time-point of observation	Observation Group	Control Group	t	P
CA125(U/mL)	Pre-treatment	322.95±37.40	327.64±41.86	0.590	0.557
	Post-treatment*	143.40±22.91	224.63±30.76	14.974	<0.001
CA153(U/mL)	Pre-treatment	121.72±3.28	121.97±4.14	0.340	0.735
	Post-treatment*	53.77±4.09	66.47±4.85	14.154	<0.001
HE4(pmol/L)	Pre-treatment	259.44±3.17	258.90±3.21	0.846	0.399
	Post-treatment*	131.32±3.64	153.39±3.72	30.020	<0.001
CD3+ (%)	Pre-treatment	42.78±5.90	43.10±6.12	0.266	0.791
	Post-treatment	48.74±5.83	43.55±6.04	4.373	<0.001
CD4+ (%)	Pre-treatment	27.26±3.76	27.99±3.80	0.966	0.336
	Post-treatment*	38.18±4.12	34.46±4.50	4.308	<0.001
CD4+/CD8+ (%)	Pre-treatment	1.27±0.09	1.27±0.08	0.362	0.718
	Post-treatment*	1.74±0.16	1.53±0.15	6.762	<0.001

***Note:***

* P<0.05.

**Table-III:** Comparison of Clinical Efficacy between the 2 Groups [n(%)].

Group	n	CR	PR	SD	PD	OR
Observation Group	50	14(28.000)	22(44.00)	8(16.00)	6(12.00)	36(72.00)
Control Group	50	11(22.00)	15(30.00)	14(28.00)	10(20.00)	26(52.00)
*χ* ^2^		4.244				
*P*		0.039				

The post-treatment SS-QOL scores of two groups were significantly improved compared with pre-treatment, and the observation group showed a more significant improvement than control group (P < 0.05). Table-IV. There is no statistically significant difference in the incidence of adverse reactions between the two groups (P > 0.05). Table-V.

**Table-IV:** Comparison of Pre-and Post-Treatment SS-QOL Scores between the 2 Groups (*χ̅*±*S*).

Group	Physical Function		Language Function		Role Function		Cognitive Function	

	Pre-treatment	Post-treatment	Pre-treatment	Post-treatment	Pre-treatment	Post-treatment	Pre-treatment	Post-treatment
Observation Group	4.74±0.44	10.86±1.01	5.74±0.72	14.92±1.28	5.72±0.50	15.52±1.05	7.82±0.52	18.38±1.16
Control Group	4.60±0.67	7.50±0.89	5.56±0.79	11.46±1.13	5.58±0.67	11.40±1.01	7.60±0.64	14.36±1.10
*t*	1.232	17.677	1.191	14.367	1.184	19.951	1.885	17.776
*P*	0.221	<0.001	0.236	<0.001	0.239	<0.001	0.062	<0.001

**Table-V:** Comparison of Adverse Drug Reactions between the 2 Groups [n (%)].

Group	Marrow suppression	Gastrointestinal Reactions	Fatigue	Liver/kidney Function Impairment	Rash/Alopecia
Observation Group	14(28.00)	17(34.00)	8(16.00)	16(32.00)	9(18.00)
Control Group	15(30.0)	14(28.00)	6(12.00)	13(26.00)	6(12.00)
*χ* ^2^	0.049	0.421	0.332	0.437	0.706
*P*	0.826	0.517	0.564	0.509	0.401

## DISCUSSION

The observation group of patients in this study adopted a PDS combined with neoadjuvant chemotherapy regimen, and the results showed that the surgery and hospitalization time of the patients in this group were shorter than those control group (P<0.05), while the amount of abdominal fluid and intraoperative blood loss were lower than control group (P<0.05), indicating that this treatment regimen can shorten surgery time, reduce intraoperative bleeding, thereby reducing surgical trauma and patient hospitalization time. This is consistent with the results of multiple studies.^5^,^6^

The five years survival rate of ovarian cancer is low, with only about 1/3 of patients achieving clinical remission.^7^ Currently, the treatment of ovarian cancer often requires combination of surgery and adjuvant chemotherapy, thereby prolonging patient survival and improving quality of life. Research has shown that neoadjuvant chemotherapy can reduce the clinical staging of tumors, shrink tumor lesions, thereby increasing the chances of patients receiving PDS and achieving the goal of improving patient prognosis. Furthermore, neoadjuvant chemotherapy can effectively inhibit tumor cell activity, reduce tumor cell diffusion caused by surgical compression/pulling, facilitate the absorption of abdominal fluid, improve postoperative recovery, and improve the overall condition of patients.^8^ Multiple research results have shown that ovarian cancer patients receiving neoadjuvant chemotherapy have a better prognosis.^9^,^10^

CA125 is a highly sensitive indicator for diagnosing ovarian cancer.^11^ The concentration of CA153 increases during ovarian cancer.^12^ HE4 exhibits high expression in ovarian cancer.^13^ The results of this study showed that the levels of CA125, CA153, and HE4 in both groups were significantly reduced after treatment compared to before treatment (P<0.05), and the observation group showed more significant decrease (P<0.05), indicating that the improvement in tumor marker levels was more significant in patients treated with PDS combined with neoadjuvant chemotherapy. Previous studies have shown that neoadjuvant chemotherapy can improve immune function and reduce the risk of recurrence.^14^ The results of this study showed that the levels of CD3+, CD4+, and CD4+/CD8+in both groups were significantly improved compared to before treatment (P<0.05), and the observation group showed more significant improvement than control group (P<0.05), indicating that the combination of PDS and neoadjuvant chemotherapy has a stronger effect on improving immune function indicators in ovarian cancer patients. The reason is that two-three cycles of neoadjuvant chemotherapy are administered to patients before surgery, which suppresses the spread ability of tumor cells, reduces the size of the lesion, facilitates surgical resection, and also reduces the risk of iatrogenic spread and recurrence, which is beneficial for improving the immune function of ovarian cancer patients.^15^

The improvement of the patient’s better quality of life is the main aspect of evaluating tumor treatment. The results of this study showed that the SS-QOL scores of observation group after treatment were higher than control group (P<0.05), But the incidence of adverse reactions is comparable to that of control group (P>0.05). This indicates that PDS combined with neoadjuvant chemotherapy is safe and reliable in the treatment of ovarian cancer, and can significantly improve the quality of life of patients. This is consistent with the results of multiple studies.^16^,^17^

### Limitations:

It includes a small number of observations and a short follow-up time. Therefore, it is necessary to expand the sample size and increase the follow-up time for further evaluation of the clinical efficacy of PDS combined with neoadjuvant chemotherapy in treating ovarian cancer.

## CONCLUSIONS

The application of PDS combined with neoadjuvant chemotherapy in treating ovarian cancer is a safe and effective approach. It can significantly shorten the operation time and hospital stay, improve the levels of serum CA125, CA153, and HE4, and enhance the immune function and quality of life for patients, which is worthy of promotion and application in clinical practice.

### Authors’ Contributions:

**HY** and **WW:** Carried out the studies, participated in collecting data, literature search, and drafted the manuscript, and are responsible and accountable for the accuracy or integrity of the work.

**SW,** **CY LZ:** Performed the statistical analysis, participated in its design and critical review.

Performed the statistical analysis and participated in its design.

All authors have read and approved the final manuscript.
